# Maternal and family factors and child eating pathology: risk and protective relationships

**DOI:** 10.1186/2050-2974-2-11

**Published:** 2014-04-29

**Authors:** Karina L Allen, Lisa Y Gibson, Neil J McLean, Elizabeth A Davis, Susan M Byrne

**Affiliations:** 1 Telethon Institute for Child Health Research, Centre for Child Health Research, The University of Western Australia, PO Box 855, West Perth, WA, Australia; 2 School of Psychology, The University of Western Australia, Crawley, WA, Australia; 3 Department of Endocrinology and Diabetes, Princess Margaret Hospital for Children, Subiaco, Australia

**Keywords:** Eating disorders, Children, Loss of control eating, Family, Risk factors, Protective factors, Running head, Family factors and eating pathology

## Abstract

**Background:**

Previous studies have found associations between maternal and family factors and child eating disorder symptoms. However, it is not clear whether family factors predict eating disorder symptoms specifically, or relate to more general child psychopathology, of which eating disorder symptoms may be one component. This study aimed to identify maternal and family factors that may predict increases or decreases in child eating disorder symptoms over time, accounting for children’s body mass index z-scores and levels of general psychological distress.

**Methods:**

Participants were 221 mother-child dyads from the Childhood Growth and Development Study, a prospective cohort study in Western Australia. Participants were assessed at baseline, 1-year follow-up and 2-year follow-up using interview and self-report measures. Children had a mean age of 10 years at baseline and 46% were male. Linear mixed models and generalised estimating equations were used to identify predictors of children’s eating disorder symptoms, with outcome variables including a global index of eating disorder psychopathology, levels of dietary restraint, levels of emotional eating, and the presence of loss of control (‘binge’) eating.

**Results:**

Children of mothers with a current or past eating disorder reported significantly higher levels of global eating disorder symptoms and emotional eating than other children, and mothers with a current or past eating disorder reported significantly more concern about their children’s weight than other mothers. Maternal concern about child weight, rather than maternal eating disorder symptoms, was significant in predicting child eating disorder symptoms over time. Family exposure to stress and low maternal education were additional risk factors for eating disorder symptoms, whilst child-reported family satisfaction was a protective factor.

**Conclusions:**

After adjusting for relevant confounding variables, maternal concern about child weight, children’s level of family satisfaction, family exposure to stress, and maternal education are unique predictors of child eating disorder symptoms.

## Background

It is recognised that families do not cause eating disorders [1] and there is robust evidence to support a strong genetic component to these conditions [2]. Nonetheless, certain family characteristics may increase risk for eating disorders and/or problematic eating behaviours in childhood and adolescence (e.g., [3-5]). This study sought to extend previous research by elucidating risk and protective factors in the relationships between family variables and eating disorder symptoms in children, with attention to the specificity of any identified effects.

Previous research has focused on two main categories of family characteristics, including maternal eating disorder history, maternal eating disorder symptoms and a family emphasis on eating, weight and shape; and general family functioning and parenting practices, which incorporate family conflict, family support and the frequency and nature of parent–child interactions. Moreover, studies have considered two main categories of child eating behaviour, including feeding patterns and difficulties in infancy and early childhood; and eating, weight and shape concerns, dieting, binge eating and purging in later childhood and adolescence. The following sections review key findings across these areas.

### Maternal and family emphasis on eating, weight and shape

Available data suggest that mothers with an eating disorder history are more likely to have infants with feeding difficulties, to restrict their child’s eating in early and middle childhood, and to use food for non-nutritive purposes (e.g., reward, distraction) when compared to mothers without an eating disorder history [6-11]. Mothers with an eating disorder history may also be more likely to experience concern about their child’s eating and weight, and to have children who themselves report elevated concerns about eating, weight and shape [6,12]. There is some evidence that these effects are gender-specific, with female children of mothers with an eating disorder history being affected to a greater degree than male children [6]. There is little evidence for associations between maternal eating disorder history and children’s body mass index (BMI), self-esteem, or parent-reported emotional and behavioural problems [12].

Maternal and/or familial emphasis on eating, weight and shape (e.g., parental dieting, comments about weight and shape in the home environment) may also predict weight loss attempts, weight and shape concerns, binge eating and purging in children and adolescents. Data suggest that these links apply cross-sectionally [13,14] and longitudinally [15-20], but may only occur when other predisposing factors (such as neuroticism or a general vulnerability to psychological difficulties) are present in children [21] or mothers [22]. Again, there appear to be gender differences in the strength of associations between variables, with girls, in particular, more likely to experience (or perceive) family-based pressures regarding eating or weight than boys [23-25].

Results in this area are consistent with sociocultural theories of eating disorders, which view internalisation of the ‘thin ideal’ and/or perceived pressure to be thin as key contributors to body dissatisfaction and eating pathology [26]. In childhood and adolescence, the family environment is an important source of information about cultural values and ideals. Mothers with an eating disorder history, or current eating disorder symptoms, may also find it difficult to know when and how to feed their children, or doubt their judgement in this area [10]. As noted by others [10,27,28], it is likely that these family environmental factors work in conjunction with genetic vulnerability to increase eating disorder risk.

### Family functioning and relationships

The second category of research has focused on associations between family functioning and parenting style (e.g., frequency of family interactions, family conflict) and child and adolescent eating disorder symptoms. Several groups have found prospective associations between low family affection or high family conflict and later onset of eating disorder symptoms [5,29-31] or increasing weight and shape concerns [32] in male and female adolescents. It is generally accepted that child eating disorder symptoms are likely to impact deleteriously on family interactions, meaning that cross-sectional links between family conflict and eating pathology are difficult to interpret [1]. However, parent-adolescent connectedness (i.e., the presence of parents at home, parental knowledge of their adolescent’s behaviour) and frequent family communication have been identified as significant, cross-sectional protective factors for adolescent dieting and eating disorder symptoms [33]. Greater parent-adolescent connectedness and greater family meal frequency have also been identified as significant prospective protective factors for dieting, binge eating and purging in adolescent girls [31,34].

These findings suggest that a positive family environment may help to reduce vulnerability to eating disorder symptoms in adolescence. This is consistent with the broader literature on links between family support and adolescent resilience and coping [35], and the potential for positive environmental factors to help negate risks associated with negative environmental exposures and/or underlying genetic vulnerabilities [36].

### The specificity of identified risk effects

Overall, there is evidence for associations between certain family characteristics and both feeding behaviour in early childhood, and eating disorder symptoms in later childhood and adolescence. Less is known about the specificity of these associations. In the case of maternal eating disorder symptoms, it is important to distinguish between the effects of maternal eating pathology and those of more general psychiatric morbidity. Research from the Avon Longitudinal Study of Parents and Children (ALSPAC) [11] helps to address this issue. In the ALSPAC sample (n = 12,050), women with a self-reported history of anorexia nervosa (AN; n = 247) or bulimia nervosa (BN; n = 194) reported more feeding difficulties in the first 12 months post-partum than general control participants [12]. In most cases, however, these feeding difficulties were comparable to those reported by ASLPAC women with a non-eating related psychiatric disorder (‘psychiatric control participants’) (n = 1,148). This suggests that feeding difficulties in infancy may be associated with general maternal psychiatric distress, rather than eating disorders specifically. This possibility was supported by follow-up analyses in the ALSPAC cohort, which showed that associations between maternal eating disorder history and infant feeding difficulties were mediated by maternal anxiety and depressive symptoms [28]. A recent publication extends these infant findings to child emotional and behaviour problems at 3 to 4 years of age [37]. The ALSPAC mothers with an eating disorder history in pregnancy reported greater emotional and behavioural difficulties in their children at 3.5 years, compared to control mothers, but these associations were largely accounted for by anxiety and depressive symptoms during pregnancy. The direct effect of a maternal eating disorder history on child functioning was small [37].

These results highlight that maternal eating disorder history may be a proxy for other relevant maternal or family factors, rather than a strong specific predictor of child eating pathology. Comparing children of mothers with an eating disorder history to children of mothers with a history of another, non-eating related psychiatric disorder is important if the effects of maternal eating pathology are to be fully understood. Further, any effects of eating pathology should be evaluated in the context of general maternal psychological distress (e.g., depressive and anxiety symptoms).

It is also important to determine if family characteristics predict child eating disorder symptoms specifically, or are associated with general psychological distress, of which eating disorder symptoms may be one component. This is particularly true for studies on family functioning, which is likely to have a diverse influence on offspring development [35]. To address this, studies need to consider if family functioning predicts offspring eating disorder symptoms after children’s general psychological functioning has been accounted for.

### The current study

This study sought to extend previous research by examining risk and protective relationships between family characteristics (including maternal eating disorder history, maternal eating disorder symptoms, family emphasis on eating, weight and shape, and indices of family functioning) and child eating behaviours (early feeding practices and later eating disorder symptoms), with attention to the specificity of any identified effects. Children were followed from pre to early adolescence, a high risk period for the emergence of eating disorder symptoms, and early feeding practices were assessed retrospectively.

Specific aims were as follows:

Aim 1. To compare children of mothers with a current or past eating disorder (‘eating disorder group’), children of mothers with a non-eating related current or past psychiatric disorder (‘psychiatric control group’), and children of mothers with no psychiatric history (‘general control group’) on early feeding practices, child eating disorder symptoms, and child psychological well-being.

Aim 2. To identify family characteristics that can predict increases or decreases in child eating disorder symptoms over time, taking into account child body mass and general psychological distress.

The following hypotheses were tested:

Hyp. 1. Children in the maternal eating disorder group would score significantly higher on measures of early feeding difficulties (shorter breastfeeding duration, early commencement of solids) and eating disorder symptoms (overall eating, weight and shape concern, dieting, emotional eating, binge eating) than children in the general and psychiatric control groups (Aim 1).

Hyp. 2. Maternal anxiety and depressive symptoms would be stronger predictors of increases in child eating disorder symptoms over time than maternal eating disorder history or maternal eating disorder symptoms (Aim 2).

Hyp. 3. Positive family interactions (assessed through parent and child report) would protect against increases in child eating disorder symptoms over time (Aim 2).

## Methods

### Design

This research involved data from the Childhood Growth and Development (GAD) Study. The GAD Study is a prospective cohort study based in Perth, Western Australia, which has a central focus on weight and eating behaviour in childhood. Children were recruited from Perth primary schools and interviewed at baseline (Time 1), 1-year follow-up (Time 2), and 2-year follow-up (Time 3). Additional details of the GAD Study methodology, including detailed recruitment information, have been reported previously [38-40].

### Participants

Participants were community-recruited mother-child dyads where the child was aged between 8 and 13 years at baseline and where baseline, 1-year follow-up and 2-year follow-up assessments were completed between January 2004 and January 2009. This equated to 221 children (46% male) and, due to 55 sibling pairs or triplets, 166 mothers. Family clustering was accounted for in analyses. There were 15 father-child dyads who were excluded from analyses, due to the small number of participating fathers and the focus on maternal characteristics in analyses.

Participant attrition over the 2-year period was 32% (baseline N = 325 children). Differences between families included in this study and those lost to follow-up are discussed under Preliminary Analyses, below.

At the baseline assessment, children had a mean age of 10.78 years (SD 1.72, range = 8-13 years) and mothers had a mean age of 40.98 years (SD 5.17, range = 30-54 years). As the mean age of the sample exceeded 12 years at Time 3, pubertal stage was considered as a possible confounder of the results. This was assessed via self-report Tanner stages [41], which have been found to converge well with physical examination in pre-adolescent samples [42,43]. All boys were pre-pubertal at baseline and 93% (n *=* 95/102) remained pre-pubertal at 2-year follow-up. There were no significant differences between pre- and post-pubertal boys at 2-year follow-up, for any of the assessed variables. Most girls (94%; n *=* 112/119) were pre-pubertal at baseline and 72% (n *=* 86/119) were pre-pubertal at 2-year follow-up. Girls who were post-pubertal were significantly older than girls who were not at both time points, but pubertal status did not relate significantly to the family variables of interest or children’s eating disorder symptoms. Thus, pubertal status was not included as a covariate in analyses.

### Procedure

Children and mothers were assessed separately by trained GAD Study researchers, at baseline, 1-year follow-up, and 2-year follow-up. Assessments were conducted in the family’s own home, at the Telethon Institute for Child Health Research or at Princess Margaret Hospital for Children, according to family preference. With the exception of the GAD Study interview questions and the Eating Disorder Examination (EDE; [44]), all maternal measures were completed as self-report questionnaires. In contrast, all child measures were administered verbally. The same measures were administered at all assessment points.

All GAD Study researchers received comprehensive training prior to conducting assessments, and had Honours level qualifications or above in psychology, medicine or exercise physiology. Training for the EDE and ChEDE was conducted by the first and last authors, who have extensive experience with the administration of these interviews. The first author also checked all EDE/ChEDE scores prior to data entry, to ensure adherence to scoring protocols.

Ethics approval was provided by the Ethics Committee of Princess Margaret Hospital for Children. Mothers provided written informed consent for participation.

### Outcome variables: child feeding problems and eating disorder symptoms

#### Child feeding problems (maternal report)

Questions about early feeding practices were included in the GAD Study parent interview. Specifically, two questions were used to determine the age at which mothers stopped breastfeeding their child(ren) (to the nearest week; 0 weeks if they did not breastfeed at all) and the age at which they started their child(ren) on solid food (to the nearest month).

### Child eating disorder symptoms (child report)

Child eating disorder symptoms were assessed using the Child Eating Disorder Examination (ChEDE) [45] and Children’s Affect Regulation Scale (CARES) [46].

The ChEDE is a semi-structured interview adapted from the adult Eating Disorder Examination (EDE) [44]. Like the adult version, it generates four subscale scores (Restraint, Eating Concern, Weight Concern, Shape Concern) and a Global score (the mean of the four subscale scores). A Brief Global score appears to be a more reliable index of eating pathology than the original four subscales, which are not supported by factor analysis [47,48]. The brief scale consists of eight items from the original Weight Concern and Shape Concern scales, and the resulting index correlates very highly with the original Global score and moderately to highly with each of the original four subscales [47,48]. This study used the Brief Global score as an index of overall child eating disorder psychopathology (α = .88 at baseline). To allow a specific focus on dietary restraint, we also made use of the ChEDE Restraint scale, which consists of 5 items regarding actual and intended dieting behaviour (α at baseline = .69).

The ChEDE also allows children to be categorised according to whether or not they experienced loss of control or ‘binge’ eating. Loss of control eating encompasses objective binge eating (feeling out of control of one’s eating and consuming an objectively large amount of food) and subjective binge eating (feeling out of control of one’s eating but not consuming a large amount of food). It is established that loss of control, regardless of the amount of food eaten, is the key component of binge eating behaviour in childhood [49] and objective and subjective binge eating were both considered for this research.

The CARES was used to assess emotional eating. The measure includes 10 items that assess the tendency to eat as a means of affect regulation. Scores can range from 0 (no emotional eating) to 4 (very frequent emotional eating). The alpha coefficient was .82 at baseline.

### Predictor variables: family characteristics

#### Maternal eating disorder and psychiatric disorder history (maternal report)

Five questions in the GAD Study parent interview were used to determine if mothers (i) had ever had an eating disorder or been suspected of having one and, if yes, (ii) the nature of the disorder, (iii) when it occurred, (iv) if it was diagnosed, and (v) if it was treated.

Five additional questions in the GAD Study parent interview were used to determine if mothers (i) had ever experienced an emotional or psychological problem not related to eating and, if yes, (ii) the nature of the problem/s, (iii) when they occurred, (iv) if they were diagnosed, and (v) if they were treated.

Responses to these items were used to categorise mothers according to whether they had a past eating disorder history, a non-eating related current or past psychiatric disorder, or no history of a psychiatric disorder.

### Current maternal eating disorder symptoms (maternal report)

The adult EDE version 12 was used to assess current maternal eating disorder symptoms. As per the ChEDE, this semi-structured interview generates four subscale scores (Restraint, Eating Concern, Weight Concern, Shape Concern) and a Global score (the mean of the four subscale scores), and includes diagnostic items for determining eating disorder diagnoses. The measure is established as reliable and valid [50-53]. Again, there is greater support for the Brief Global scale than the original subscales [47]. This study used the Brief Global score as an index of overall maternal eating disorder psychopathology (α = .85 at baseline). Diagnostic items were used to determine current *DSM-5* eating disorder diagnoses in mothers.

### Maternal concern about child weight (maternal report)

One question in the GAD Study parent interview was used to assess mother’s level of concern about their child(ren)’s weight, with the available response options being “not at all”, “a little”, “moderately” or “very” concerned. If the mother had multiple children participating in the study, separate ratings were made for each. As very few mothers reported being “very” concerned, responses in the two highest categories were collapsed to give a moderate-high concern group.

An additional question was used to assess mother’s level of concern about their child(ren)’s overall health. Mothers rated their child(ren)’s current physical health on a 5-point scale ranging from “poor” (1) to “excellent” (5).

### Family relationships (maternal and child report)

Family relationships were assessed in two ways. First, mothers completed the self-report General Functioning Scale (GFS) of the McMaster Family Functioning Device [54]. The GFS includes 12 items regarding family relationships, arguments and interactions, and has been established as reliable and valid with a variety of clinical and non-clinical groups [54-57]. Higher scores indicate greater family conflict and disharmony. The alpha coefficient in this sample was .90 at baseline.

Second, the Family Satisfaction subscale of the Students’ Life Satisfaction Scale (SLSS; [58]) was verbally administered to children. This subscale is designed to assess children’s level of satisfaction with their family, parents, and the nature of family interactions. It contains seven items, and subscale scores can range from 0 to 5; higher scores indicate greater family satisfaction. The measure has been established as reliable and valid [58-60], and the alpha coefficient in this sample was .78 at baseline.

### Parenting style (maternal report)

The Parenting Scale [61] was used to assess for unhelpful parenting practices. The scale includes 30 items regarding overly permissive discipline (“Laxness”), inappropriate displays of anger, meanness or irritability (“Overreactivity”), and the use of lengthy verbal responses or instructions even when ineffective (“Verbosity”). It can be used to generate subscale scores for each of these categories of items, as well as an overall global score. In each instance, scores can range from one to seven, with higher scores indicating less positive parenting. The measure has acceptable psychometric properties, although the factor structure of the three subscales has been debated [62,63]. Global scores were used in this study and the 30 items were internally consistent (α = .78).

### Family exposure to stressful life events (maternal report)

The List of Threatening Experiences [64], otherwise known as the Life Events Scale, was used to assess family exposure to stressful life events. The scale asks respondents to indicate if they have experienced each of 12 negative life events (e.g., unemployment, significant illness) over the past 6 months, with scores calculated by summing the number of “yes” responses. It has been established as a valid index of life stress [65-67].

### Maternal depressive, anxiety and stress symptoms (maternal report)

The 21-item self-report Depression Anxiety Stress Scale (DASS) [68] was used to assess maternal depressive, anxiety and stress symptoms experienced over the previous week. The DASS generates three subscale scores (Depression, Anxiety and Stress) that can range from 0 to 42. It has been established as reliable and valid [68-72]. Scores on the Depression subscale correlate highly with scores on other measures of depression, and scores on the Anxiety subscale correlate highly with those on other measures of anxiety [68,69,71]. Alpha coefficients were satisfactory in this sample (=.77 - .86).

### Maternal self-esteem (maternal report)

The Rosenberg Self-Esteem Scale (RSES) [73] was used to assess overall maternal self-esteem. The scale includes 10 items and scores can range from 10 to 40, with higher scores indicating greater self-esteem. It has well-established psychometric properties [73-75]. The alpha coefficient at baseline was .88.

### Covariates and additional descriptive variables

#### Child psychosocial functioning (child report)

Indices of child psychosocial functioning were considered as potential confounders of associations between family characteristics and child eating disorder symptoms.

The short form of the Child Depression Inventory (CDI) [76] was used to assess depressive symptoms. The CDI is a modification of the Beck Depression Inventory, and is the most commonly used instrument for assessing depressive symptoms in children. The short-form CDI contains 10 items that assess negative mood, anhedonia, ineffectiveness, and negative self-esteem, and it has been established as internally consistent and successfully used to screen children for depression [76]. It was internally consistent in this sample (α = .73 at baseline).

The Global Self-Worth subscale of the Self-Perception Profile for Children (SPPC) [77] was used to assess children’s overall or global level of self-esteem. The SPPC is a multi-dimensional measure of self-esteem and has been validated for use with participants aged eight years and older [77,78]. The alpha coefficient for the Global Self-Worth scale at baseline was .71.

The Multidimensional Media Influences Scale (MMIS) [79] was used to assess children’s awareness and internalisation of the thin-ideal, and their perceived level of media pressure to achieve the thin ideal. It has acceptable psychometric properties [79] and the total score was used in this study (α = .75).

### Family sociodemographic information (maternal report)

Questions in the GAD Study parent interview were used to assess total family income, mother’s marital status, mother’s highest level of education and mother’s general health, for consideration as covariates in analyses.

For family income, a dichotomous variable was created to reflect whether or not the family could be considered to have a low income relative to Australian standards. “Low income” was defined as an annual pre-tax income in the lowest population quartile, which equated to approximately $35,000 per annum. For marital status, a dichotomous variable was created to reflect whether or not the mother was married or in an equivalent long-term de facto relationship. For education, a dichotomous variable was created to reflect whether or not the mother had completed high school. For maternal health, mothers rated their current physical health on a 5-point scale ranging from “poor” (1) to “excellent” (5).

### Maternal and child Body Mass Index (BMI)

Height and weight were measured during the GAD Study interview. For mothers, BMI was calculated using the standard formula (weight [kg]/height [m]^2^). For children, age- and sex-specific BMI z-scores were calculated using the CDC 2000 reference data [80].

### Statistical analyses

Responses to the EDE and GAD Study interview questions were used to categorise mothers into one of three groups: an *eating disorder group*, if they met criteria for a current eating disorder on the EDE or had a previous eating disorder as diagnosed by a health professional; a *psychiatric control group*, if they had a current or previous non-eating related psychiatric disorder as diagnosed by a health professional; or a *general control group*, if there was no evidence of a current or past eating or psychiatric disorder. Groups were compared on maternal and family factors.

To address Aim 1, linear mixed models were constructed to examine the effects of maternal group (eating disorder vs. psychiatric control vs. general control) on early child feeding practices (breastfeeding duration, age of introduction of solids), child eating disorder symptoms, child psychological functioning, general child health, and level of maternal concern about child weight. Mixed linear models allow grouping variables (i.e., family status) to be controlled for, meaning that any effects of clustering of children within families could be accounted for in the analyses. These models considered the effects of maternal group on early child feeding practices as reported at a single time point (retrospectively assessed at baseline) and childhood variables as assessed annually from baseline to 2-year follow-up. Support for Hypothesis 1 would come from children of mothers with a past or current eating disorder showing greater eating disorder symptoms, on average, over the 2-year study period than children of mothers in the psychiatric and general control groups.

To address Aim 2, linear mixed models and generalised estimating equations were used to identify significant predictors of changes in child eating disorder symptoms over time. Continuous outcome variables (for use in linear mixed models) included ChEDE Brief Global scores and Restraint scores, and CARES emotional eating scores. The categorical outcome variable (for use in generalised estimating equations) was loss of control eating. Time invariant and time-varying family factors were considered as predictor variables and child BMI z-score was included as a covariate in all multivariate models.

*Time invariant predictors* were those variables assessed at baseline only, or which showed considerable stability over the 2 year assessment period and so were used at baseline only. These included maternal group (eating disorder vs. psychiatric control vs. general control) and level of maternal concern about child weight (no concern vs. slight concern vs. moderate-high concern) as family predictor variables, and family income (low vs. medium-high), mother’s marital status (married/de facto vs. no long-term relationship) and maternal education (high school vs. less than high school) as potential covariates. For these variables, associations were between the predictor variable at baseline and average levels of the outcome variable over the 2 year study period. A significant interaction between a time invariant predictor and time would show that the predictor variable at baseline predicted changes in the outcome variable over the subsequent 2 years.

*Time-varying predictors* were those variables that were assessed annually from baseline to 2-year follow-up, and which showed variation over the assessment period. These included scores for the EDE Brief Global scale, DASS, RSES, Life Event Scale, Parenting Scale, McMaster Family Functioning scale and SLSS, as family predictor variables, as well as maternal BMI as a potential covariate. For these variables, associations were between changes in the predictor variable over the 2 year study period and changes in the outcome variable over the same time frame.

Predictors were initially examined in univariate models, and those that were significant at *p* < .05 were later entered in multivariate analyses. As note, all multivariate models included child BMI z-score. In addition, a more complex model was specified that included child BMI z-score as well as child depressive symptoms and global self-esteem. If family factors predicted child eating disorder symptoms after depressive symptoms and self-esteem were accounted for, evidence would be provided for a specific association between family factors and child eating disorder symptoms.

Models were constructed for boys and girls together, with gender interaction terms used to explore possible gender differences in the degree or nature of associations between variables. Consistent with recommendations, analyses were initially conducted using non-interaction variables only and subsequently conducted using non-interaction and interaction terms [81]. Interaction terms were only retained if they contributed to a model significantly.

Support for Hypothesis 2 would come from mother’s DASS scores being significant longitudinal predictors of increasing child eating disorder symptoms. Support for Hypothesis 3 would come from mother’s McMaster Family Functioning scores and/or children’s SLSS family satisfaction scores being significant longitudinal predictors of decreasing eating disorder symptoms.

## Results

### Preliminary analyses

Consistent with other longitudinal studies, GAD Study families lost to follow-up tended to be more socially disadvantaged at baseline than families who remained in the study. Specifically, mothers lost to follow-up (n = 100) were less likely to have completed high school (*p* = .002), more likely to be single or divorced (*p* = .041), and more likely to report family exposure to stressful live events (*p* = .010) at baseline than mothers included in analyses (n = 166). However, there were no significant baseline differences between children lost to follow-up (n = 104) and those included in this study (n = 221) on any of the child variables assessed, including eating disorder symptoms.

Of the 166 mothers included in analyses, eight met criteria for a current *DSM-5* eating disorder. Of these, five women met criteria for binge eating disorder (BED), two met criteria for bulimia nervosa (BN) and one met criteria for anorexia nervosa (AN). A further 10 women were determined to have a history of an eating disorder, and had previously been diagnosed with AN (n *=* 5) or an AN-like disorder (n = 2), BN (n = 2), or AN and BN at different time points (n *=* 1). These 10 participants and the eight current eating disorder cases were included in the maternal eating disorder group (n *=* 18; 11% of the maternal sample).

Of the women who did not meet criteria for a current or past eating disorder, 36 (22%) reported experiencing a current or past emotional problem that had warranted diagnosis and/or treatment. In most cases, the problem was depression (with or without anxiety; n *=* 28) and was treated with medication (n *=* 25). Other reported problems included generalised anxiety (without depression; n = 2), post-traumatic stress disorder (n *=* 2), substance abuse/dependence (n *=* 1), and agoraphobia (n *=* 1). These 36 participants were included in the maternal psychiatric disorder group. A further 10 women reported experiencing a current or past emotional problem that had not been diagnosed or treated. Due to difficulties in validating the nature and severity of these problems, these participants were not included in the psychiatric disorder group, and were instead excluded from analyses.

The 102 mothers (61%) who did not report a current or past eating disorder or psychiatric disorder were included in the general (non-psychiatric) control group.

Baseline demographic details and mean scores on maternal and family-based measures are shown in Table 1 by maternal group. Significant between-group differences are also shown, as identified using a one-way ANOVA (for continuous variables) and chi square tests (for categorical variables).

**Table 1:** Maternal characteristics (mean [SD] unless otherwise indicated) at baseline, by maternal psychiatric group (Ns refer to number of mothers)

	**General control (n = 102)**	**Psychiatric control (n = 36)**	**Eating disorder (n = 18)**
Age	41.38 (5.52)	41.50 (5.39)	38.90 (6.17)
% married or de facto	73.5% (75/102)	58.3% (21/36)	66.7% (12/18)
% low family income	5.9% (6/102)_a_	22.2% (8/36)_b_	0% (0/0)_a_
% completed high school	61.8% (63/102)_a_	66.7% (24/36)_a_	27.8% (5/18)_b_
BMI	25.83 (5.33)	24.97 (4.37)	28.11 (6.47)
Maternal health	3.59 (0.92)_a_	3.54 (1.01)	3.07 (1.03)_b_
EDE brief global	0.82 (0.83)_a_	0.92 (1.35)_a_	2.05 (1.57)_b_
DASS depression	1.22 (1.48)_a_	2.65 (3.95)	6.57 (5.53)_b_
DASS anxiety	1.01 (1.35)_a_	2.00 (2.58)	4.80 (4.61)_b_
DASS stress	3.87 (3.01)	5.56 (3.87)	5.80 (6.34)
Rosenberg self-esteem scale	32.96 (3.82)_a_	30.50 (4.70)_b_	28.33 (5.85)_b_
Family stress – life events scale	1.38 (1.53)	1.45 (1.78)	2.20 (2.70)
Parenting scale	3.03 (0.53)	3.11 (0.58)	3.12 (0.60)
McMaster family functioning scale	1.68 (0.41)	1.59 (0.44)	1.91 (0.26)

*Note.* Columns with different subscripts are significantly different at *p* < .05 as identified through analysis of variance (continuous variables) or Chi square (categorical variables) comparisons.

BMI = Body Mass Index; EDE = Eating Disorder Examination; DASS = Depression Anxiety Stress Scale.

### Aim 1: Effects of maternal eating disorder history on child eating behaviour and general psychological functioning

Linear mixed model analyses identified significant main effects of maternal group (eating disorder vs. psychiatric control vs. general control) on the age at which solids were introduced, *F*(2, 512) = 5.65, *p* = .004, degree of maternal concern about child weight, *F*(2, 507) = 7.79, *p* < .001, ChEDE Brief Global scores, *F*(2, 558) = 3.30, *p* = .038, and child emotional eating *F*(2, 562) = 3.27, *p* = .039, after controlling for the clustering of children within families. Children born to mothers with an eating disorder history were given solids significantly earlier, had significantly higher levels of eating disorder psychopathology (on average from baseline to 2-year follow-up), and were significantly more likely to report emotional eating (on average from baseline to 2-year follow-up) than children born to mothers in the psychiatric disorder and general control groups (see Table 2). In addition, there was a significant main effect of maternal group on the likelihood of mothers reporting slight concern, Wald χ^2^ (2) = 6.94, *p* = .031, or moderate-high concern, Wald χ^2^ (2) = 14.91, *p* = .001, about their child’s weight. Follow-up analyses revealed that mothers in the eating disorder group were more concerned about their child’s weight than mothers in the general control group, but did not differ significantly from mothers in the psychiatric control group (see Table 2).

**Table 2:** Child characteristics (mean [SE] unless indicated) at baseline, by maternal psychiatric group (Ns refer to number of children)

	**No eating disorder/psychiatric history (n = 154)**	**Psychiatric history (n = 49)**	**Eating disorder history (n = 18)**
Child BMI z-score	0.67 (0.08)	0.74 (0.14)	1.18 (0.28)
Child gender – % male	47.4% (73/ 154)	44.9% (22/ 49)	54.5% (10/ 18)
Maternal report variables			
Age started solids (weeks)	21.86 (0.49)_a_	20.40 (0.88)_a_	15.96 (1.79)_b_
Age stopped breastfeeding (weeks)	39.75 (1.66)	36.70 (2.97)	36.09 (6.25)
Child health	4.04 (0.07)	3.95 (0.13)	3.91 (0.25)
Concern for child weight			
% no concern	77.9% (120/154)_a_	59.2% (29/49)	27.8% (5/18)_b_
% slight concern	14.9% (23/154)_a_	26.5% (13/49)	44.4% (8/18)_b_
% moderate-high concern	7.1% (11/154)_a_	14.3% (7/49)	27.8% (5/18)_b_
Child report variables			
ChEDE brief global	0.52 (0.07)_a_	0.42 (0.13)_a_	1.02 (0.27)_b_
ChEDE restraint	0.24 (0.53)	0.26 (0.65)	0.40 (0.78)
Loss of control eating – %	7.8% (12/ 154)	6.1% (3/ 49)	11.1% (2/ 18)
CARES	0.46 (0.04)_a_	0.51 (0.08)_a_	0.78 (0.15)_b_
CDI	1.77 (0.22)	1.73 (0.41)	2.45 (0.82)
SPPC global	3.32 (0.05)	3.41 (0.09)	3.08 (0.17)
MMIS	1.57 (0.42)	1.51 (0.39)	1.77 (0.46)
SLSS	4.17 (0.06)	4.21 (0.10)	3.96 (0.20)

*Note.* Columns with different subscripts are significantly different at *p* < .05 as identified through linear mixed models (continuous variables) or generalised estimating equations (categorical variables). Effects apply at baseline and when considering average scores over the full 2-year study period.

BMI = Body Mass Index, ChEDE = Child Eating Disorder Examination, CARES = Children’s Affect Regulation Scale, CDI = Child Depression Inventory, SPPC = Self-Perception Profile for Children, MMIS = Multidimensional Media Influences Scale, SLSS = Student’s Life Satisfaction Scale.

There were no significant effects of maternal group on the age at which breastfeeding stopped, ChEDE Restraint scores, loss of control eating, CDI scores, or SLSS scores (*p*s = .081 - .645). There were trend level, though non-significant, effects of maternal group on child BMI z-score (*p* = .051), SPPC Global self-esteem scores (*p* = .052) and MMIS scores (*p* = .057).

Table 2 summarises baseline information for each of the dependent variables and the identified between-group effects.

### Aim 2: Maternal and family predictors of child eating disorder symptoms

#### ChEDE Brief Global score

Parenting Scale scores, family exposure to stressful life events, maternal depressive, anxiety and stress symptoms, and children’s SLSS family satisfaction scores were identified as significant time-varying predictors of ChEDE Brief Global scores in univariate analyses. Baseline maternal concern about child weight and maternal education were identified as significant time invariant predictors. There were no significant interaction effects between time and concern about child weight or between time and maternal education.

When the above variables were entered in a multivariate model with child BMI z-score as a covariate, DASS depression scores, SLSS family satisfaction scores, maternal education, and maternal concern about child weight contributed to the model significantly (see Table 3, Model 1). When the multivariate model was re-run with child depressive symptom and self-esteem scores as additional covariates (Table 3, Model 2), maternal education and maternal concern about child weight continued to predict eating disorder symptoms, but maternal depression and children’s family satisfaction did not. Model coefficients showed that ChEDE Brief Global scores were 0.18 points lower, on average over the 2-year period, when mothers had completed high school relative to when they had not. Brief Global scores were 0.64 points higher, on average over the 2-year period, when maternal concern about child weight was moderate-high at baseline relative to minimal (no concern) at baseline. There were no significant interaction effects with child sex (see Table 3).

**Table 3:** Multivariate models for the longitudinal relationship between maternal and family variables and child eating disorder symptoms

	**Model 1**		**Model 2**	
*ChEDE brief global*	Coefficient (95% CI)	*p*	Coefficient (95% CI)	*p*
Parenting scale	0.01 (-0.12, 0.14)	.875	-0.03 (-0.16, 0.10)	.636
Life events scale	0.01 (-0.04, 0.07)	.562	0.02 (-0.03, 0.07)	.402
DASS depression	0.02 (0.01, 0.04)	.033*	0.02 (-0.01, 0.04)	.094
DASS anxiety	-0.01 (-0.03, 0.01)	.431	-0.02 (-0.04, 0.01)	.121
DASS stress	-0.01 (-0.02, 0.02)	.833	0.01 (-0.01, 0.02)	.645
SLSS family satisfaction	-0.27 (-0.38, -0.16)	<.001**	-0.05 (-0.17, 0.07)	.408
Maternal education (ref: completed high school)				
< High school	0.19 (0.03, 0.35)	.021*	0.18 (0.02, 0.33)	.023*
Concern about child weight (ref: no concern)				
Slight concern	0.01 (-0.19, 0.21)	.931	-.01 (-0.20, 0.18)	.908
Moderate-high concern	0.67 (0.40, 0.93)	<.001**	0.64 (0.39, 0.88)	<.001**
*ChEDE restraint scale*	Coefficient (95% CI)	*p*	Coefficient (95% CI)	*p*
Parenting scale	0.10 (0.02, 0.18)	.021*	0.07 (-0.01, 0.16)	.100
Concern about child weight (ref: no concern)				
Slight concern	-0.01 (-0.13, 0.13)	.968	-0.02 (-0.16, 0.11)	.745
Moderate-high concern	0.27 (0.11, 0.44)	.001**	0.25 (0.08, 0.41)	.003**
*CARES emotional eating*	Coefficient (95% CI)	*p*	Coefficient (95% CI)	*p*
Life events scale	0.05 (0.02, 0.08)	.001**	0.04 (0.01, 0.07)	.017*
DASS depression	0.01 (-0.01, 0.02)	.056	0.01 (-0.01, 0.02)	.292
*Loss of control eating*	Odds ratio (95% CI)	*p*	Odds ratio (95% CI)	*p*
DASS anxiety	1.06 (1.02, 1.14)	.150	1.03 (0.95, 1.12)	.470
SLSS family satisfaction	0.44 (0.29, 0.72)	<.001**	0.56 (0.35, 0.90)	.026*

*Note.* Models include variables that predicted the outcome significantly in univariate analyses. Model 1 is adjusted for child body mass index (BMI) z-score only. Model 2 is adjusted for child BMI z-score and child depressive symptom and global self-esteem scores. Coefficients show the predicted change in the dependent variable per unit change in the predictor variable, as identified through linear mixed models. Generalised estimating equations were used to determine odds ratios for the categorical outcome of loss of control eating.

*p < .05 **p < .001.

### ChEDE Restraint score

Parenting Scale scores were the only significant time-varying predictor of ChEDE Restraint scores in univariate analyses, and baseline maternal concern about child weight was the only significant time invariant predictor. The interaction between time and concern about child weight was not significant.

When Parenting Scale scores and maternal concern about child weight were entered in a multivariate model with child BMI z-score as a covariate, both remained significant in predicting ChEDE Restraint scores (see Table 3, Model 1). When the multivariate model was re-run with child depressive symptoms and self-esteem scores as additional covariates (Table 3, Model 2), maternal concern about child weight remained significant in predicting ChEDE Restraint but Parenting Scale scores did not. Model coefficients showed that ChEDE Restraint scores were 0.25 points higher, on average over the 2-year period, when maternal concern about child weight was moderate-high at baseline relative to minimal (no concern) at baseline. There were no significant interaction effects with child sex (see Table 3).

### Emotional eating

Family exposure to stressful life events and maternal depressive symptoms were identified as significant time-varying predictors of emotional eating in univariate analyses. There were no significant time invariant predictors of emotional eating.

When family stress and maternal depressive symptoms were entered in a multivariate model with child BMI z-score as a covariate, family stress was significant in predicting emotional eating scores in children (Table 3, Model 1). This relationship remained significant when child depressive symptoms and self-esteem scores were added as covariates (Table 3, Model 2). Model coefficients showed that for every stressful event experienced over the 2-year study period, CARES emotional eating scores may be expected to increase by 0.04 over the same time frame. There were no significant interaction effects with child sex (see Table 3).

### Loss of control eating

Maternal anxiety symptoms and children’s SLSS family satisfaction scores were identified as significant time-varying predictors of loss of control eating in univariate analyses. There were no significant time invariant predictors of loss of control eating.

When maternal anxiety symptoms and children’s family satisfaction scores were entered in a multivariate model with child BMI z-score as a covariate, family satisfaction was significant in predicting loss of control eating (Table 3, Model 1). This relationship remained significant when child depressive symptoms and self-esteem scores were added as covariates (Table 3, Model 2). A 1 point increase in family satisfaction scores over time (equivalent to moving from mildly to moderately satisfied) was associated with a 44% reduction in risk for loss of control eating, although there was some uncertainty around this estimate with 95% confidence intervals suggesting a 10% to 65% reduction in risk (see Table 3). Again, there were no significant interaction effects with child sex.

## Discussion

This study aimed to determine whether eating disorder symptoms and psychological well-being would differ significantly across children of mothers with an eating disorder history, children of mothers with a history of another psychiatric disorder, and children of mothers with no past or present eating or psychiatric disorder. In addition, it aimed to identify family factors that could predict increases or decreases in child eating disorder symptoms over time, with attention to the specificity of any identified effects. In final multivariate models, maternal education and maternal concern about child weight were significant in predicting general eating disorder psychopathology; maternal concern about child weight was significant in predicting dietary restraint; family exposure to stressful life events was significant in predicting emotional eating; and children’s family satisfaction scores were significant in predicting loss of control eating. Results did not differ significantly across boys and girls.

Partial support was provided for Hypothesis 1. Children in the maternal eating disorder group did score significantly higher on the Brief Global index of eating disorder psychopathology and the CARES index of emotional eating than children in the general and psychiatric control groups. Mothers with a past or present eating disorder also introduced solids significantly earlier than other mothers, and were more concerned about their children’s weight. These results are consistent with previous findings (e.g., [6,12]), and were specific to children of mothers with an eating disorder history: children of mothers with another psychiatric disorder were not affected. We found no significant between-group differences for dietary restraint or rates of loss of control eating in children. This may reflect a genuine lack of association between maternal disorders and these offspring behaviours, or stem from the relatively small number of mothers with a past or present eating disorder, combined with relatively low rates of dieting and loss of control eating amongst children. Stein et al. [12] did find higher rates of dieting, as well as heightened weight and shape concerns, in their sample of 10-year-old children born to mothers with an eating disorder. Thus, further research may be required to determine whether dieting is consistently inflated amongst children of mothers past or present eating disorders.

The second phase of analyses provides insight into the mechanisms through which maternal eating disorder history may influence child eating behaviour. Contrary to Hypothesis Two, maternal depressive and anxiety symptoms did not predict child eating disorder symptoms. Maternal eating disorder history and maternal eating disorder symptoms also failed to predict child eating pathology. Instead, the most robust predictor in multivariate models was maternal concern about child weight. In almost all cases (>90%), this concern was in relation to perceived child overweight rather than underweight. Parent-perceived child overweight also emerged as a significant risk factor for adolescent eating disorders in a separate Australian study [82], lending support to the generalizability of these findings. In this study, it deserves note that maternal concern about child weight was relatively stable across the study period, and baseline levels did not predict subsequent *changes* in child eating disorder symptoms. Instead, baseline maternal concern was significant in predicting children’s eating disorder symptoms on average over the next 2 years. Children of mothers who were concerned about their weight at baseline had consistently high levels of weight and shape concern and dietary restraint over the next 2 years.

Maternal education was an additional baseline predictor of Brief Global ChEDE scores, such that children of mothers who had not completed high school had higher levels of eating disorder psychopathology across the 2-year study period than children of mothers who had completed high school. Maternal education may be a marker of general socio-demographic status, whereby children from more disadvantaged or ‘resource poor’ families are more vulnerable to heightened weight and shape concerns. Alternatively, mothers who do not complete high school may differ from other mothers in their capacity to model healthy eating and body image messages, and/or in the emphasis they place on weight and shape. Further research is needed to address these possibilities, and to elucidate the mechanisms through which maternal education may influence child eating disorder psychopathology. It is interesting to note that, in this sample, media influences regarding the thin-ideal were not significant in predicting children’s eating disorder symptoms. This suggests that family factors may be more important than media influences in accounting for eating disorder features in this age group. With adolescent samples, some studies have found family, peer and personality factors to be more important than media influences in predicting eating disorder symptoms [83,84], whilst other data suggest that family and media influences are more important than peer factors [16]. Regardless of age, it seems important that studies on media influences and eating pathology also attend to family predictors.

Two variables predicted changes in child eating disorder symptoms over time. Consistent with Hypothesis 3, child-reported family satisfaction was a significant protective factor for loss of control eating, with increases in SLSS scores over time predicting decreases in vulnerability to loss of control eating. This relationship applied even after child BMI z-score and general psychological distress were accounted for. Increases in SLSS scores over time were also significant in predicting decreases in children’s Brief Global ChEDE scores over the same time period, but this relationship ceased to reach statistical significance after adjusting for child psychological distress. These results highlight the potential benefits of family support in protecting against child and adolescent eating disorder symptoms, particularly loss of control eating. Research with adolescents has also linked positive family relationships to lower rates of adolescent dieting [31,34], a finding not observed here. This difference may stem from cross-study variation in the age of participants (late childhood / early adolescence for the current sample, versus later adolescence in prior reports) and/or in the assessment approach used. In this research, the ChEDE was used to assess a mix of dietary restriction (actual restriction of eating) and restraint (intent to restrict eating) over the past month. Previous studies have tended to rely on single item assessing self-reported dieting frequency in the past year [31,34], which may capture a different type of behaviour. Ongoing attention to the potential protective benefits of positive family relationships, and their impact on different eating disorder symptoms over adolescence, seems warranted.

Family stress was also a significant longitudinal predictor of emotional eating. The magnitude of this effect was small, with 25 stressful events in 2 years being required to predict an increase in emotional eating from “sometimes” to “often” on the CARES scale. Nonetheless, emotional eating may be understood as a coping mechanism for children who are exposed to repeated or pronounced stressful events over time. Exposure to stressful life events has also been linked to the development of binge eating disorder [85], and found to precipitate and maintain emotional eating in adolescent [86] and adult [87] samples. To our knowledge, this is the first study to identify a prospective association between family exposure to stress and increases in emotional eating in childhood.

There are four main implications arising from these results. First, maternal concern about child weight appears to be a strong predictor of child eating disorder symptoms, even after objective body weight and general child psychological distress are adjusted for. Thus, interventions designed to prevent or reduce child eating pathology may benefit from targeting mother’s concerns about, and attitudes towards, their children’s weight. This may need to occur early in childhood as, in the current study, mothers’ levels of concern about their child(ren)’s weight were already stable, tracking from middle childhood onwards.

Second, family satisfaction may be a protective factor for loss of control eating. The transition to adolescence is a high risk time for eating disorder onset and a period of changing family dynamics, with children starting to individuate and develop an identity autonomous from their family. Working to enhance family support and relationships during this period may be beneficial for eating disorder prevention.

Third, family stress and low maternal education may increase risk for emotional eating and weight and shape concerns, respectively. Accordingly, children who experience repeated exposure to family stress and children of mothers who did not complete high school education may benefit from additional support during their adolescent years. As noted, there is also a need for more research to identify the mechanisms through which low maternal education may influence child eating disorder symptoms.

Fourth, maternal eating disorder symptoms and general maternal psychological distress do not predict child eating disorder symptoms when other family and child variables are accounted for. This is important, because these features have traditionally been viewed as risk factors for child eating pathology and may distract from family variables that genuinely do serve as a marker of risk.

This study has a number of strengths. The use of interview-based measures to assess eating disorder symptoms provides support for the validity of the data, and the longitudinal design allows issues of causality to be considered. Additional advantages include the use of general and psychiatric control participants; the use of mother and child report data; and consideration of gender differences in the effects of maternal and family factors. The study also has limitations, including the reliance on self-report data for determining maternal psychiatric disorders and past maternal eating disorders, the small number of mothers in the eating disorder group, participant attrition over time, and the use of single-item measures for some constructs. Although all mothers stated that they had been diagnosed or treated for a psychiatric condition or eating disorder, information on the validity of those diagnoses was not available. Moreover, women who did not wish to disclose a psychiatric history, and those who did not seek diagnosis or treatment for psychiatric difficulties, would not have been identified in this study. If larger group sizes were available, it would be beneficial for past and current maternal eating disorders to be distinguished, along with different eating disorder diagnoses. Finally, families with lower maternal education and greater exposure to stress at baseline were more likely to be lost to follow-up. As maternal education and family stress predicted eating disorder symptoms in children, efforts should be made to try and retain families with these characteristics in future studies of this nature. For the current research, effect estimates for these predictor variables may be conservative in nature.

## Conclusions

This study has provided insight into associations between maternal and family factors and child eating disorder symptoms, with attention to the relative importance of different maternal and family predictors and the specificity of identified effects. Consistent with previous findings, children of mothers with a current or past eating disorder reported significantly higher levels of some eating disorder symptoms than other children, and mothers with a current or past eating disorder reported significantly more concern about their children’s weight than other mothers. Maternal concern about child weight, rather than maternal eating disorder symptoms, was significant in predicting child eating disorder symptoms. Other family variables of importance included children’s level of family satisfaction, family exposure to stress, and maternal education.

## Abbreviations

ALSPAC: Avon longitudinal study of parents and children; BMI: Body mass index; CARES: Children’s affect regulation scale; ChEDE: Child eating disorder examination; CDI: Child depression inventory; DASS: Depression anxiety stress scale; EDE: Eating disorder examination; GAD Study: Childhood growth and development study; MoBa: Norwegian Mother and Child Cohort Study; MMIS: Multidimensional media influences scale; RSES: Rosenberg self-esteem scale; SLSS: Students’ life satisfaction scale; SPPC: Self-perception profile for children.

## Competing interests

The authors declare that they have no competing interests.

## Authors’ contributions

ED and SB contributed to the design of the Childhood Growth and Development Study. KA, LG, ED and SB collected data for the Childhood Growth and Development Study. KA, NM and SB designed the current research study. KA performed the statistical analyses and drafted the manuscript. All authors read and approved the final manuscript.

## References

[B1] Le Grange D Lock J Loeb KL Nicholls D Academy for eating disorders position paper: the role of the family in eating disorders Int J Eat Disord 2010 43 1 5 19728372 10.1002/eat.20751

[B2] Campbell IC Mill J Uher R Schmidt U Eating disorders, gene-environment interactions and epigenetics Neurosci Biobehav Rev 2011 35 784 793 10.1016/j.neubiorev.2010.09.012 20888360

[B3] Patel P Wheatcroft R Park RJ Stein A The children of mothers with eating disorders Clin Child Fam Psychol Rev 2002 5 1 19 10.1023/A:1014524207660 11993543

[B4] Schmidt U Humfress H Treasure J The role of general family environment and sexual and physical abuse in the origins of eating disorders Eur Eat Disord Rev 1997 5 184 207

[B5] Johnson JG Cohen P Kasen S Brook JS Childhood adversities associated with risk for eating disorders or weight problems during adolescence or early adulthood Am J Psychiatry 2002 159 394 400 10.1176/appi.ajp.159.3.394 11870002

[B6] Agras WS Hammer LD McNicholas F A prospective study of the influence of eating-disordered mothers on their children Int J Eat Disord 1999 25 253 262 10.1002/(SICI)1098-108X(199904)25:3<253::AID-EAT2>3.0.CO;2-Z 10191989

[B7] Stice E Agras SW Hammer LD Risk factors for the emergence of childhood eating disturbances: a five-year prospective study Int J Eat Disord 1999 25 375 387 10.1002/(SICI)1098-108X(199905)25:4<375::AID-EAT2>3.0.CO;2-K 10202648

[B8] Koubaa S Hällstrom T Hirschberg AL Early maternal adjustment in women with eating disorders Int J Eat Disord 2008 41 405 410 10.1002/eat.20521 18306346

[B9] Waugh E Bulik CM Offspring of women with eating disorders Int J Eat Disord 1999 25 123 133 10.1002/(SICI)1098-108X(199903)25:2<123::AID-EAT1>3.0.CO;2-B 10065389

[B10] Reba-Harreleson L Von Holle A Hamer RM Torgersen L Reichborn-Kjennerud T Bulik CM Patterns of maternal feeding and child eating associated with eating disorders in the Norwegian mother and child cohort study (MoBa) Eat Behav 2010 11 54 61 10.1016/j.eatbeh.2009.09.004 19962121 PMC2790429

[B11] Micali N Simonoff E Treasure J Infant feeding and weight in the first year of life in babies of women with eating disorders J Pediatr 2009 154 55 60 10.1016/j.jpeds.2008.07.003 18783793

[B12] Stein A Woolley H Cooper S Winterbottom J Fairburn CG Cortina-Borja M Eating habits and attitudes among 10-year-old children of mothers with eating disorders: longitudinal study Br J Psychiatry 2006 189 324 329 10.1192/bjp.bp.105.014316 17012655 PMC1888733

[B13] Hill AJ Weaver C Blundell JE Dieting concerns of 10-year-old girls and their mothers Br J Clin Psychol 1990 29 346 348 10.1111/j.2044-8260.1990.tb00894.x 2252953

[B14] Edmunds H Hill AJ Dieting and the family context of eating in young adolescent children Int J Eat Disord 1999 25 435 440 10.1002/(SICI)1098-108X(199905)25:4<435::AID-EAT8>3.0.CO;2-3 10202654

[B15] Linville D Stice E Gau J O'Neil M Predictive effects of mother and peer influences on increases in adolescent eating disorder risk factors and symptoms: a 3-year longitudinal study Int J Eat Disord 2011 44 745 751 10.1002/eat.20907 21344465 PMC4053444

[B16] Field AE Camargo CAJ Taylor CB Berkey CS Roberts SB Colditz GA Peer, parent and media influences on the development of weight concerns and frequent dieting among preadolescent and adolescent girls and boys Pediatrics 2001 107 54 60 10.1542/peds.107.1.54 11134434

[B17] McCabe MP Ricciardelli LA A prospective study of pressures from parents, peers, and the media on extreme weight change behaviors among adolescent boys and girls Behav Res Ther 2005 43 653 668 10.1016/j.brat.2004.05.004 15865919

[B18] Field AE Javaras KM Aneja P Kitos N Camargo CA Taylor CB Laird NM Family, peer, and media predictors of becoming eating disordered Arch Pediatr Adolesc Med 2008 162 574 579 10.1001/archpedi.162.6.574 18524749 PMC3652375

[B19] Helfert S Warschburger P A prospective study on the impact of peer and parental pressure on body dissatisfaction in adolescent girls and boys Body Image 2011 8 101 109 10.1016/j.bodyim.2011.01.004 21354379

[B20] Neumark-Sztainer D Bauer KW Friend S Hannan PJ Story M Berge JM Family weight talk and dieting: how much do they matter for body dissatisfaction and disordered eating behaviors in adolescent girls? J Adolesc Health 2010 47 270 276 10.1016/j.jadohealth.2010.02.001 20708566 PMC2921129

[B21] Davis C Shuster B Blackmore E Fox J Looking good- family focus on appearance and the risk for eating disorders Int J Eat Disord 2004 35 136 144 10.1002/eat.10250 14994350

[B22] Farrow CV Blissett J Do obsessive compulsive symptoms mediate the relationship between maternal eating psychopathology and restrictive feeding practices? Int J Eat Disord 2009 42 76 80 10.1002/eat.20579 18704918

[B23] Smolak L Levine MP Schermer F Parental input and weight concerns among elementary school children Int J Eat Disord 1999 25 263 271 10.1002/(SICI)1098-108X(199904)25:3<263::AID-EAT3>3.0.CO;2-V 10191990

[B24] Peterson KA Paulson SE Williams KK Relations of eating disorder symptomology with perceptions of pressures from mother, peers, and media in adolescent girls and boys Sex Roles 2007 56 629 639 10.1007/s11199-007-9207-3

[B25] Phares V Steinberg AR Thompson JK Gender differences in peer and parental Influences: body image disturbance, self-worth, and psychological functioning in preadolescent children J Youth Adolesc 2004 33 421 429

[B26] Stice E Risk and maintenance factors for eating pathology: a meta-analytic review Psychol Bull 2002 128 825 848 12206196 10.1037/0033-2909.128.5.825

[B27] Reichborn-Kjennerud T Bulik CM Tambs K Harris JR Genetic and environmental influences on binge eating in the absence of compensatory behaviors: a population-based twin study Int J Eat Disord 2004 36 307 314 10.1002/eat.20047 15478129

[B28] Micali N Simonoff E Stahl D Treasure J Maternal eating disorders and infant feeding difficulties: maternal and child mediators in a longitudinal general population study J Child Psychol Psychiatry 2011 52 800 807 10.1111/j.1469-7610.2010.02341.x 21073463

[B29] Beato-Fernandez L Rodriguez-Cano T Belmonte-Llario A Martinez-Delgado C Risk factors for eating disorders in adolescents: a Spanish community-based longitudinal study Eur Child Adolesc Psychiatry 2004 13 287 294 10.1007/s00787-004-0407-x 15490276

[B30] Moorhead DJ Stashwick CK Reinherz HZ Giaconia RM Streigel-Moore RM Paradis AD Child and adolescent predictors for eating disorders in a community population of young adult women Int J Eat Disord 2003 33 1 9 10.1002/eat.10105 12474194

[B31] Berge JM Wall M Larson N Eisenberg ME Loth KA Neumark-Sztainer D The unique and additive associations of family functioning and parenting practices with disordered eating behaviors in diverse adolescents J Behav Med 2014 37 205 217 10.1007/s10865-012-9478-1 23196919 PMC3605198

[B32] May AL Kim JY McHale SM Crouter AC Parent-adolescent relationships and the development of weight concerns from early to late adolescence Int J Eat Disord 2006 39 729 740 10.1002/eat.20285 16927386

[B33] Fonseca H Ireland M Resnick MD Familial correlates of extreme weight control behaviors among adolescents Int J Eat Disord 2002 32 441 448 10.1002/eat.10078 12386908

[B34] Haines J Kleinman KP Rifas-Shiman SL Field AE Austin B Examination of shared risk and protective factors for overweight and disordered eating among adolescents Arch Pediatr Adolesc Med 2010 164 336 343 20368486 10.1001/archpediatrics.2010.19 PMC3093706

[B35] Viner RM Ozer EM Denny S Marmot M Resnick M Fatusi A Currie C Adolescence and the social determinants of health Lancet 2012 379 1641 1652 10.1016/S0140-6736(12)60149-4 22538179

[B36] Belsky J Pluess M Beyond diathesis stress: differential susceptibility to environmetal influences Psychol Bull 2009 135 885 908 19883141 10.1037/a0017376

[B37] Micali N Stahl D Treasure J Simonoff E Childhood psychopathology in children of women with eating disorders: understanding risk mechanisms J Child Psychol Psychiatry 2013 2013 In press 10.1111/jcpp.12112 PMC4217387 23808622

[B38] Allen KL Byrne SM Blair EM Davis EA Why do some overweight children experience psychological problems? The role of weight and shape concern Int J Pediatr Obes 2006 1 239 247 10.1080/17477160600913552 17907331

[B39] Allen KL Byrne SM McLean NJ Davis EA Overconcern with weight and shape is not the same as body dissatisfaction: evidence from a prospective study of pre-adolescent boys and girls Body Image 2008 5 261 270 10.1016/j.bodyim.2008.03.005 18585990

[B40] Gibson LY Byrne SM Jacoby P Davis E The role of family and maternal factors in childhood obesity Med J Aust 2007 186 591 595 17547550 10.5694/j.1326-5377.2007.tb01061.x

[B41] Tanner JM Growth at adolescence 1962 Springfield: Charles C Thomas

[B42] Brooks-Gunn J Warren MP Rosso J Gargiulo J Validity of self-report measures of girls’ pubertal status Child Dev 1987 58 829 841 10.2307/1130220 3608653

[B43] Duke PM Lit IF Gross RT Adolescents’ self-assessment of sexual maturation Pediatrics 1980 66 918 920 7454482

[B44] Fairburn CG Cooper Z Fairburn CG, Wilson GT The Eating Disorder Examination Binge eating: Nature, assessment and treatment 1993 12 New York: Guilford Press 317 360

[B45] Bryant-Waugh RJ Cooper PJ Taylor CL Lask BD The use of the eating disorder examination with children: a pilot study Int J Eat Disord 1996 19 391 397 10.1002/(SICI)1098-108X(199605)19:4<391::AID-EAT6>3.0.CO;2-G 8859397

[B46] Allen KL Byrne SM McLean NJ The dual-pathway and cognitive-behavioural models of binge eating: prospective evaluation and comparison Eur Child Adolesc Psychiatry 2012 21 51 62 10.1007/s00787-011-0231-z 22120762

[B47] Byrne SM Allen KL Lampard AM Dove ER Fursland A The factor structure of the eating disorder examination in clinical and community samples Int J Eat Disord 2010 43 260 265 19350647 10.1002/eat.20681

[B48] Jongenelis MI Byrne SM Pettigrew S Allen KL Watts F A psychometric examination of a modified 8-item version of the children’s eating disorder examination Psychol Assess 2014 In press 10.1037/a0034803 24188154

[B49] Tanofsky-Kraff M Marcus MD Yanovski SZ Yanovski JA Loss of control eating disorder in children age 12 years and younger: proposed research criteria Eat Behav 2008 9 257 380 10.1016/j.eatbeh.2007.09.002 18549996 PMC2495768

[B50] Luce KH Crowther JH The reliability of the eating disorder examination-self-report questionnaire version (EDE-Q) Int J Eat Disord 1999 25 349 351 10.1002/(SICI)1098-108X(199904)25:3<349::AID-EAT15>3.0.CO;2-M 10192002

[B51] Grilo CM Masheb RM Lozano-Blanco C Barry DT Reliability of the eating disorder examination in patients with binge eating disorder Int J Eat Disord 2004 35 80 85 10.1002/eat.10238 14705160

[B52] Guest T Using the eating disorder examination in the assessment of bulimia and anorexia: issues of reliability and validity Soc Work Health Care 2000 31 71 83 10.1300/J010v31n04_05 11140344

[B53] Rizvi SL Peterson CB Crow SJ Agras SW Test-retest reliability of the eating disorder examination Int J Eat Disord 2000 28 311 316 10.1002/1098-108X(200011)28:3<311::AID-EAT8>3.0.CO;2-K 10942917

[B54] Epstein NB Baldwin LM Bishop DS The McMaster family assessment device J Marital Fam Ther 1983 9 171 180

[B55] Georgiades K Boyle MH Jenkins JM Sanford M Lipman E A multilevel analysis of whole family functioning using the McMaster family assessment device J Fam Psychol 2008 22 344 354 18540763 10.1037/0893-3200.22.3.344

[B56] Byles J Byrne C Boyle MH Offord DR Ontario child health study: reliability and validity of the general functioning subscale of the McMaster family assessment device Fam Process 1988 27 97 104 10.1111/j.1545-5300.1988.00097.x 3360100

[B57] McDermott BM Batik M Roberts L Gibbon P Parent and child report of family functioning in a clinical child and adolescent eating disorders sample Aust N Z J Psychiatry 2002 36 509 514 10.1046/j.1440-1614.2002.01043.x 12169151

[B58] Heubner ES Preliminary development and validation of a multidimensional life satisfaction scale for children Psychol Assess 1994 6 149 158

[B59] Huebner E Laughlin JE Ash C Gilman R Further validation of the multidimensional students’ life satisfaction scale J Psychoeduc Assess 1998 16 118 134 10.1177/073428299801600202

[B60] Greenspoon PJ Saklofske DH Validity and reliability of the multidimensional students’ life satisfaction scale with Canadian children J Psychoeduc Assess 1997 15 138 155 10.1177/073428299701500204

[B61] Arnold DS O'Leary SG Wolff LS Acker MM The parenting scale: a measure of dysfunctional parenting in discipline situations Psychol Assess 1993 5 137 144

[B62] Arney F Rogers H Baghurst P Sawyer M Prior M The reliability and validity of the parenting scale for Australian mothers of preschool-aged children Aust J Psychol 2008 60 44 52 10.1080/00049530701458076

[B63] Rhoades KA O'Leary SG Factor structure and validity of the parenting scale J Clin Child Adolesc Psychol 2007 36 137 146 10.1080/15374410701274157 17484687

[B64] Brugha T Bebbington P Tennant C Hurry J The list of threatening experiences: a subset of 12 life event categories with considerable long-term contextual threat Psychol Med 1985 15 189 194 10.1017/S003329170002105X 3991833

[B65] Wagner C Abela JR Brozina K A comparison of stress measures in children and adolescents: a self-report checklist versus an objectively rated interview J Psychopathol Behav Assess 2006 28 251 261

[B66] Brugha T Cragg D The list of threatening experiences: the reliability and validity of a brief life events questionnaire Acta Psychiatr Scand 1990 82 77 81 10.1111/j.1600-0447.1990.tb01360.x 2399824

[B67] McQuaid JR Monroe SM Roberts JE Johnson SL Garamoni DJ Kupfer DJ Frank E Toward the standardization of life stress assessment: definitional discrepancies and inconsistencies in methods Stress Med 1992 8 47 56 10.1002/smi.2460080107

[B68] Lovibond SH Lovibond PF Manual for the Depression Anxiety Stress Scales 1995 2 Sydney: Psychology Foundation

[B69] Antony MM Bieling PJ Cox BJ Enns MW Swinson RP Psychometric properties of the 42-item and 21-item versions of the depression anxiety stress scales in clinical groups and a community sample Psychol Assess 1998 10 176 181

[B70] Gloster AT Rhoades HM Novy D Klotsche J Senior A Kunik M Wilson N Stanley MA Psychometric properties of the depression anxiety and stress scale-21 in older primary care patients J Affect Disord 2008 110 248 259 10.1016/j.jad.2008.01.023 18304648 PMC2709995

[B71] Henry JD Crawford JR The short-form versions of the depression anxiety stress scales (DASS-21): construct validity and normative data in a large non-clinical sample Br J Clin Psychol 2005 44 227 239 10.1348/014466505X29657 16004657

[B72] Norton PJ Depression anxiety and stress scales (DASS-21): psychometric analysis across four racial groups Anxiety Stress Coping 2007 20 253 265 10.1080/10615800701309279 17999228

[B73] Rosenberg M Society and the adolescent self-image (revised) 1989 Princeton, CT: Princeton University Press

[B74] Robins RW Hendin HM Trzesniewski KH Measuring global self-esteem: construct validation of a single-item measure and the rosenberg self-esteem scale Pers Soc Psychol Bull 2001 27 151 161 10.1177/0146167201272002

[B75] Shevlin ME Bunting BP Lewis CA Confirmatory factor analysis of the Rosenberg self-esteem scale Psychol Rep 1995 76 707 710 10.2466/pr0.1995.76.3.707

[B76] Kovacs M The Child Depression Inventory (CDI) manual 1992 Toronto, Canada: Multi Health Systems

[B77] Harter S Manual for the Self-Perception Profile for Children 1985 Denver, CO: University of Denver

[B78] Muris P Meesters C Fijen P The self-perception profile for children: further evidence for its factor structure, reliability, and validity Pers Individ Dif 2003 35 1791 1802 10.1016/S0191-8869(03)00004-7

[B79] Cusumano DL Thompson JK Media influence and body image in 8–11-year-old boys and girls: a preliminary report on the multidimensional media influence scale Int J Eat Disord 2001 29 37 44 10.1002/1098-108X(200101)29:1<37::AID-EAT6>3.0.CO;2-G 11135331

[B80] Ogden CL Kuczmarski RJ Flegal KM Mei Z Guo S Wei R Grummer-Strawn LM Curtin LR Roche AF Johnson CL Centers for disease control and prevention 2000 growth charts for the United States: improvements to the 1977 National Center for Health Statistics version Pediatrics 2002 109 45 60 10.1542/peds.109.1.45 11773541

[B81] Aiken LS West SG Multiple regression: Testing and interpreting interactions 1991 Newbury Park, CA: Sage

[B82] Allen KL Byrne SM Forbes D Oddy WH Risk factors for full- and partial-syndrome early adolescent eating disorders: a population-based pregnancy cohort study J Am Acad Child Adolesc Psychiatry 2009 48 800 809 10.1097/CHI.0b013e3181a8136d 19564799

[B83] Ferguson CJ Munoz ME Winegard B Winegard B The influence of heritability, neuroticism, maternal warmth and media use on disordered eating behaviors: a prospective analysis of twins Psychiatr Q 2012 83 353 360 10.1007/s11126-012-9205-7 22278805

[B84] Ferguson CJ Munoz ME Garza A Galindo M Concurrent and prospective analyses of peer, television and social media influences on body dissatisfaction, eating disorder symptoms and life satisfaction in adolescent girls J Youth Adolesc 2013 In press 10.1007/s10964-012-9898-9 23344652

[B85] Striegel-Moore RH Dohm FA Kraemer HC Schreiber GB Taylor CB Daniels SR Risk factors for binge-eating disorders: an exploratory study Int J Eat Disord 2007 40 481 487 10.1002/eat.20400 17573685

[B86] Nguyen-Rodriguez ST Chou C-P Unger JB Spruijt-Metz D BMI as a moderator of perceived stress and emotional eating in adolescents Eat Behav 2008 9 238 246 10.1016/j.eatbeh.2007.09.001 18329603 PMC2386154

[B87] Adam TC Epel ES Stress, eating and the reward system Physiol Behav 2007 91 449 458 10.1016/j.physbeh.2007.04.011 17543357

